# Antimicrobial resistance and its relationship with antimicrobial use on Austrian dairy farms

**DOI:** 10.3389/fvets.2023.1225826

**Published:** 2023-07-21

**Authors:** Thomas Werner, Annemarie Käsbohrer, Barbara Wasner, Sandra Köberl-Jelovcan, Sebastian G. Vetter, Christa Egger-Danner, Klemens Fuchs, Walter Obritzhauser, Clair L. Firth

**Affiliations:** ^1^Unit of Veterinary Public Health and Epidemiology, Institute of Food Safety, Food Technology and Veterinary Public Health, University of Veterinary Medicine, Vienna, Austria; ^2^Department for Biological Safety, German Federal Institute for Risk Assessment, Berlin, Germany; ^3^Upper Austrian Animal Health Organization Laboratory, Clinical Microbiology, Upper Austrian Animal Health Organization, Ried im Innkreis, Austria; ^4^Institute for Medical Microbiology and Hygiene, Centre for Foodborne Infectious Diseases, Division of Public Health, Austrian Agency for Health and Food Safety (AGES), Graz, Austria; ^5^ZuchtData EDV-Dienstleistungen GmbH, Vienna, Austria; ^6^Data, Statistics and Risk Assessment, Austrian Agency for Health and Food Safety (AGES), Graz, Austria; ^7^Veterinary Practice, Parschlug, Austria

**Keywords:** cattle, antimicrobial resistance, antibiotics, ESBL, calves, *Escherichia coli*

## Abstract

The aim of this study was to investigate the prevalence of ESBL/AmpC-producing *E. coli* and the resistance pattern of commensal *E. coli*, as well as the link between the use of antibiotics (AMU) and the occurrence of resistance in *E. coli* on Austrian dairy farms. AMU data from 51 farms were collected over a one-year period in 2020. Fecal samples were collected from cows, pre-weaned and weaned calves in 2020 and 2022. Samples were then analyzed using non-selective and selective agar plates, *E. coli* isolates were confirmed by MALDI-TOF analysis. Broth microdilution was used for antimicrobial susceptibility testing. The AMU of each farm was quantified as the number of Defined Daily Doses (nDDD_vet_) and Defined Course Doses (nDCD_vet_) per cow and year. Cephalosporins (mean 1.049; median 0.732 DDD_vet_/cow/year) and penicillins (mean 0.667; median 0.383 DDD_vet_/cow/year) were the most frequently used antibiotics on these farms, followed by tetracyclines (mean 0.275; median 0.084 DDD_vet_/cow/year). In 2020, 26.8% of the *E. coli* isolated were resistant to at least one antibiotic class and 17.7% of the isolates were classified as multidrug resistant (≥3 antibiotic classes). Out of 198 *E. coli* isolates, 7.6% were identified as extended-spectrum/AmpC beta-lactamase (ESBL/AmpC) producing *E. coli*. In 2022, 33.7% of *E. coli* isolates showed resistance to at least one antibiotic and 20.0% of isolates displayed multidrug resistance. Furthermore, 29.5% of the samples carried ESBL/AmpC-producing *E. coli*. In 2020 and 2022, the most frequently determined antibiotic resistances among commensal *E. coli* isolates were to tetracyclines, sulfonamides and penicillins. In addition, pre-weaned calves had the highest resistance rates in both years. Statistical analyses showed a significant association between low and high use AMU classifications for penicillins (in nDDD_vet_/cow/year) and their respective resistance among commensal *E. coli* isolates in 2020 (*p* = 0.044), as well as for sulfonamide/trimethoprim (*p* = 0.010) and tetracyclines (*p* = 0.042). A trend was also noted between the total amount of antibiotics used on farm in 2020 (by nDDD_vet_/cow/year) and multidrug resistances in commensal *E. coli* isolated on farm that year (*p* = 0.067). In conclusion, the relationship between AMU and antimicrobial resistance (AMR) on dairy farms continues to be complex and difficult to quantify.

## 1. Introduction

Antimicrobial resistance (AMR) is a central issue in One Health, affecting human medicine, veterinary medicine, and the environment. In 2001, the World Health Organization (WHO) ranked AMR as one of the leading threats to global health and a review on AMR estimated that, by 2050, AMR may cause up to 10 million deaths each year (1, 2). It has also been estimated that more than 1.2 million people worldwide died from infections with antibiotic-resistant pathogens in 2019 (3). The excessive use, and sometimes misuse, of antibiotics in human medicine as well as veterinary medicine has increased the spread and development of bacterial resistance mechanisms (4). However, the strength of the link between antibiotic resistance in veterinary medicine and human medicine is still controversial (5–7).

While in some countries, such as Australia and Brazil, the use of antibiotics for growth promotion in livestock production is still allowed, in the European Union (EU) this non-therapeutic use has been banned since 2006 (8). In the EU, antibiotics for veterinary use can only be obtained from veterinarians and are not freely available to buy over-the-counter. In addition, in Austria, in order for veterinarians to dispense injectable antibiotics for use in food-producing animals, farmers must be trained members of the Austrian Animal Health Service (German: *Österreichischer Tiergesundheitsdienst*-TGD) (9). The TGD is similar to the “veterinarian-client-patient relationship (VCPR)” in the United States (10), in that it regulates the existence of a contract and emergency treatment provision between farmers and their herd veterinarians, but, in addition, it also requires annual training of both parties with respect to livestock disease and medication. Furthermore, as stated above, antibiotics (and other non-parenteral drugs) can only be dispensed to farm clients who are members of the TGD. If farmers are not specifically trained TGD members, no antibiotics (except oral products) can be dispensed to them by veterinarians (11).

In Austria, since 2015 every veterinarian must report the quantities of antibiotics dispensed to each farm for the treatment of food-producing animals annually to the Austrian Agency for Health and Food Safety (AGES) (9, 12). Veterinarians must also provide documentation of all medications dispensed and administered on the farm to the farmer, who must then keep the records for 5 years. Based on the 2021 national report for Austria, 39.1 metric tonnes of antibiotics were dispensed, of which 70.6% was used for pigs, 22.7% for cattle, 6.4% for poultry and 0.3% for other animal species (13).

The current study aimed to investigate the occurrence of antimicrobial resistance among commensal *Escherichia coli* (*E. coli*), as well as the presence of extended-spectrum beta-lactamase (ESBL) and/or AmpC beta-lactamase (AmpC) producing *E. coli* on Austrian dairy farms, and the link to antimicrobial use (AMU) on these farms. Commensal *E. coli* are an important indicator for the occurrence of antimicrobial resistance along the food chain. Furthermore, they are ubiquitous intestinal inhabitants, can acquire resistance and also be the source of AMR genes transferred horizontally to other bacteria (2, 14). ESBL/AmpC-producing *E. coli* produce enzymes, which have the ability to hydrolyse ß-lactam antibiotics, such as penicillins and cephalosporins (15).

ESBLs and pAmpCs are a public health concern as bacteria become non-susceptible to third-generation cephalosporins, resulting in increased use of last-resort antibiotics, such as carbapenems, and treatment failures (16). Domestic animals, wildlife and the environment commonly harbor ESBLs/AmpCs and are considered reservoirs and vehicles for the spread of these resistances (17). While there is still a limited understanding of the frequency of transmission of resistance between livestock and humans, and a recent study has shown that the main source of ESBL/pAmpC-producing *E. coli* carriage in humans is acquired within the community, transmission to and from non-human sources is still considered important (18).

The link between AMU and the prevalence of AMR bacteria has been discussed in a variety of studies in both human and veterinary medicine (19–23). A comprehensive analysis carried out under the supervision of EFSA confirmed that a variety of factors contribute to AMR, and that there is an association between a reduction in antimicrobial use and reduced AMR (24). Studies from several countries have shown that ESBL-producing *E. coli* are present in the feces of dairy cows and are often associated with the use of antibiotics such as cephalosporins (19, 21, 25, 26).

## 2. Materials and methods

### 2.1. Study population

In total, 51 farms from 4 federal states (Upper Austria, Styria, Burgenland and Salzburg) were included in the study. The farmers were actively recruited by participating veterinary practices. A total of 11 veterinary practices agreed to participate in the study. These practices were primarily concerned with treating cattle. Some of the veterinarians had previously been involved in prior research by this study group, and, as such, this was a convenience sample consisting of interested veterinary practitioners and farmers. The enrolment criteria for this study were a minimum herd size of 10 dairy cows, which were primarily the dual-purpose breed Austrian *Fleckvieh*. All participating veterinarians and farmers were members of the Austrian Animal Health Service (TGD, *Österreichischer Tiergesundheitsdienst*).

### 2.2. Antimicrobial use data

#### 2.2.1. Data collection and availability

In the present study, the collection period for antibiotic use data was from 1st January 2020 to 31st December 2020. The data on dispensing and use of antibiotics were obtained through a combination of paper records kept by farmers and digital records maintained by herd veterinarians. Data collected included treatments of dairy cows and calves. The documentation of AMU regarding the identification of treated animals was sometimes incomplete, it was not always possible to determine which age group received the medication. In this study, we were able to draw conclusions about the age of the treated animal based on the method of application (>95% of all AMU were not orally applied and were thus counted as being administered to cows). The number of dispensed antibiotic sprays was documented, but was not included in the quantified AMU data.

The herd data of the individual farms was used to calculate production days and replacement rates. These data were obtained from the central cattle data system (*Rinderdatenverbund*, RDV system). For this purpose, only cows (i.e., female animals, which had calved at least once) that were present on the farm in 2020 were included. The calving date of heifers (first calving) was classed as the date of entry into the dairy herd in order to avoid falsification of production days. Based on national milk recording data and the lactation number of the cows, the replacement rate (i.e., proportion of first-calving heifers) could be calculated for each herd. Out of this information, the number of production days was calculated for each farm.

All data sets collected were imported into Microsoft Excel (Microsoft Corporation, Redmond, WA, United States) and subsequently analyzed descriptively.

#### 2.2.2. Quantification of AMU

Based on the recommendations of the European Medicines Agency (EMA), the quantities of AMU were calculated as number of Defined Daily Doses (27–29).

To obtain the mass of the active antimicrobial substance in milligrams, the volume of the medicinal product was multiplied by the concentration of the product and, if necessary, a conversion factor. The conversion factor for international units (IU) or for prodrugs for certain proprietary medicinal products is listed in the EMA recommendations (30).


$$\begin{array}{c} Amount\;of\;active\;ingredient\;\left(mg\right)=amount\;of\;drug\;administered\;\left(ml\right)\\ {}*Concentration\;of\;drug\;\left(\frac{mg}{ml}\right)\\ {}*conversion\;factor \end{array}$$

The Defined Daily Doses_vet_ (DDD_vet,_ given in milligram active substance per kilogram body weight) values for the individual active ingredients were taken from the recommendations of the EMA for cattle (28). The following formula was used to calculate the number of DDD_vet_ (nDDD_vet_):


$$nDDDvet=\frac{amount\;of\;active\;ingredient\;\left(mg\right)}{DDDvet\;for\;that\;antimicrobial\;active\;ingredient\;}$$

The next step was the calculation of nDDD_vet_/cow/year for injectables and oral treatment per cow and per year for each farm, as previously described elsewhere (31). The assumed weight of 500 kg of a dairy cow was taken from the EMA guidelines (32).


$$nDDDvet\left(inj,oral\right)/cow/year=\frac{nDDDvet}{\begin{array}{l} liveweight\;\left(500kg\right)\\ {}*total\;production\;days\; \end{array}}*365$$

As intramammary and intrauterine treatments are not dosed per kilogram of liveweight, the nDDD_vet_ for these treatments was calculated per cow and year as described in the formula below.


$$nDDDvet\left(intra\right)/cow/year=\frac{nDDDvet}{\;total\;production\;days\;}*365$$

As the European Medicines Agency does not provide a Defined Daily Dose (DDD_vet_) value for long-acting dry cow treatment, but only a standardized Defined Course Dose (DCD_vet_), the number of defined course doses (nDCD_vet_) per cow per year was additionally calculated for all antibiotics. As predefined by EMA, 4 dry cow injectors are counted as 1 DCD_vet_ (28). The following formula was used to calculate the nDCD_vet_ per cow and year for dry cow treatments:


$$\begin{array}{l} nDCD\left(dry\right)vet/cow/year=\frac{Number\;of\;dry\;cow\;injectors}{4}\\ /total\;production\;days*365 \end{array}$$

To prevent an over or underestimation of the number of cows dried off with antimicrobial dry cow therapy, a correction factor with respect to the replacement rate (i.e., proportion of first-calving heifers in the herd) and the respective mean calving interval of each farm was calculated for each farm (33). The nDCD_(dry)vet_/cow/year was then multiplied by the respective correction factor for each herd.


$$\begin{array}{c} correction\;factor=\frac{calving\;interval\;\left(d\right)}{365}\\ {}*\frac{100}{100-rate\;of\;first\;calving\;\left(\%\right)} \end{array}$$

For the remaining (non-dry cow) antibiotics, the DCDvet as recommended by the EMA was used to calculated nDCD_vet_ per cow and year for this fraction of treatments.

The total nDCDvet/cow/year for each farm was made up of the sum of dry cow treatments (in nDCDvet/cow/year), with the total nDCDvet/cow/year for systemic, intramammary (non-dry cow), intrauterine and oral treatments.

For statistical analysis and graphical representation, all application routes and indications were combined analyses in the nDDDvet and nDCDvet figures.

### 2.3. Antimicrobial resistance data

#### 2.3.1. Fecal sample collection

The first sampling took place between August and October 2020 and the second sampling was carried out from February to March 2022 on all study farms. Fecal samples were collected from three groups (dairy cows, pre-weaned calves and weaned calves) on each farm during both sampling periods. The sampling was carried out by one of two authors (TW and CLF). To avoid contamination of samples and possible spread of disease, protective clothing in the form of disposable coveralls, gloves, and overshoes were used during sample collection.

On each farm, two pairs of boot swabs were collected from the alleyways of the dairy cows in freestalls or from the manure area directly behind the cows in tie-stalls. Calves were divided into two groups: pre-weaned, i.e., under 6 weeks of age; and weaned, i.e., over 6 weeks of age, and were sampled with rectal swabs with Amies transport medium (Heinz Herenz GmbH, Germany). Dependent on the number of calves present on farm, up to five rectal swabs were collected per group and farm. Swabs from each age group were pooled in the laboratory.

According to the laboratory protocol of the European Union Reference Laboratory (EURL-AR) for the detection of ESBL/AmpC-producing *E. coli* in caecal content and fresh meat samples (34), the samples were refrigerated immediately after collection (5°C ± 3°C) and sent to a cooperating laboratory within 48 h. Testing for the detection of *E. coli* and ESBL-producing *E. coli* was performed within 96 h after sample collection.

#### 2.3.2. Bacteriological investigation

**Figure 1:**
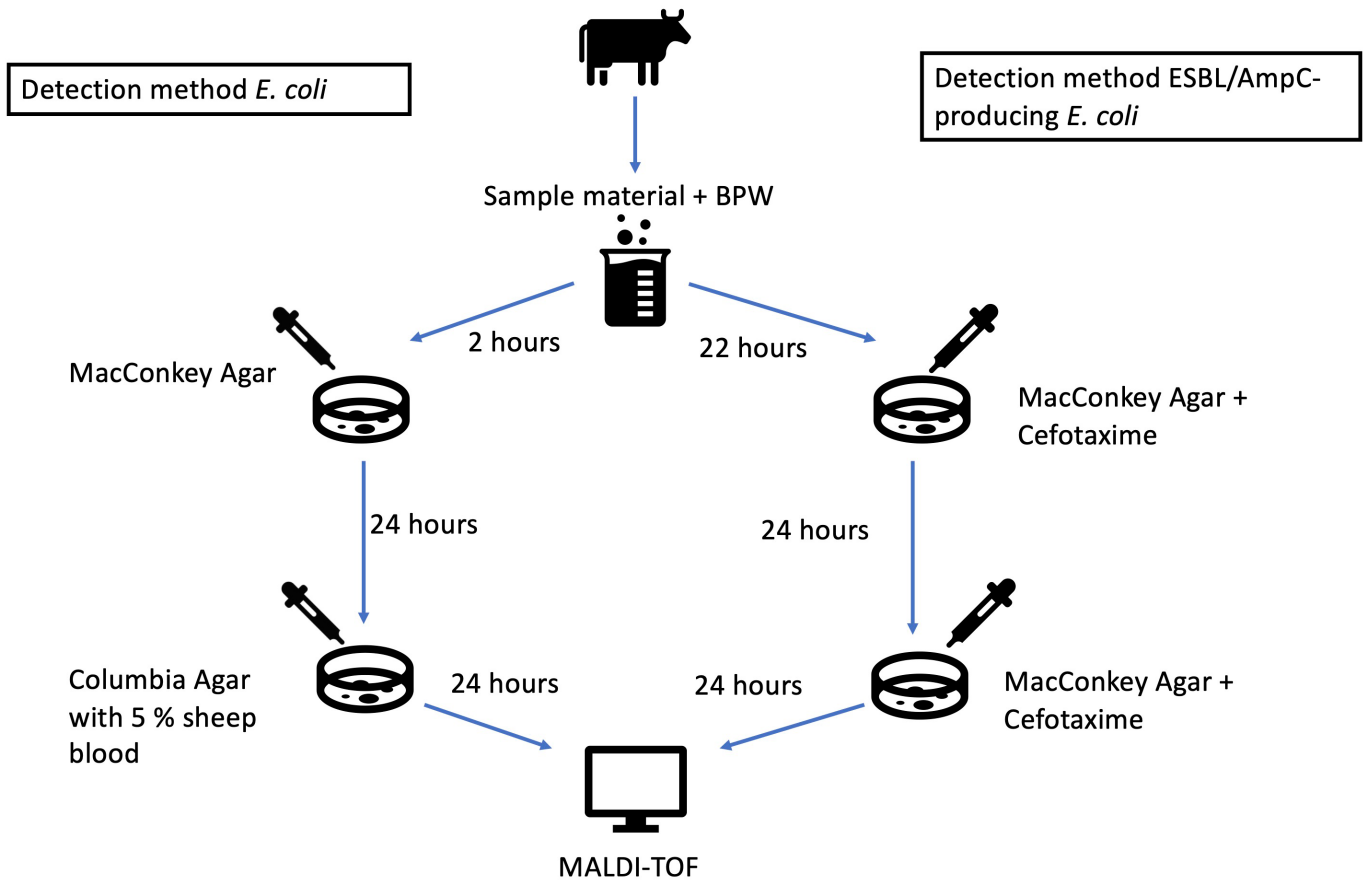
Schematic illustration for the detection of *E. coli* (left side) and ESBL/AmpC-producing *E. coli* (right side).

For the isolation of commensal *E. coli*, boot swabs were enriched in 200 mL of Buffered Peptone Water (BPW) and pooled rectal swabs were enriched in 9 mL of BPW. After 2 h of aerobic incubation at 37°C, the suspension was spread on MacConkey agar (bioMérieux, France) using a sterile 10 μL loop. The agar plates were then incubated aerobically for 24 h at 37°C ± 1°C, after which suspected colonies were inoculated onto a Columbia agar plate with 5% sheep blood (COS, bioMérieux, France). Following a further 24 h of aerobic incubation at 37°C ± 1°C, confirmation of the pure culture was performed by time-of-flight mass spectrometry (MALDI-TOF MS) (Figure 1).

For the detection of ESBL/AmpC-producing *E. coli,* the BPW suspension was incubated for 22 h at 37°C ± 1°C aerobically. A 10 μL loop of the incubated sample material was spread on a selective MacConkey agar containing 1 mg/L Cefotaxime (CTX) (MacConkey Agar + CTX, Tritium Company, The Netherlands or corresponding plate from OXOID, Germany) and incubated again aerobically at 37°C ± 1°C for 24 h. A subculture was spread on selective culture medium (MacConkey agar containing 1 mg/L CTX) and incubated again under the same conditions. The pure culture was confirmed by MALDI-TOF (Figure 1). Non-commercial MacConkey agar (Oxoid, Germany) containing 1 mg/L CTX provided by the National Reference Laboratory for Antimicrobial Resistance at the Institute for Medical Microbiology & Hygiene, Graz, was used for the second sampling in 2022.

All enrichment cultures and pure cultures were frozen in cryotubes with the addition of glycerol at −20°C.

#### 2.3.3. Antimicrobial susceptibility testing

Antimicrobial susceptibility testing was done at the National Reference Laboratory for Antimicrobial Resistance at the Institute for Medical Microbiology & Hygiene, Graz. Minimal inhibitory concentrations were determined using commercial Sensititre™ plates EUVSEC3 and EUVSEC2 from Thermo Fisher Scientific (Waltham, MA, United States) according to the manufacturer’s instructions following ISO 20776-1:2019.

Epidemiological cut-off values (ECOFFs) were used for evaluation according to the guidelines of the European Committee on Antimicrobial Susceptibility Testing (EUCAST) and the Commission Implementing Decision (EU) 2020/1729 (Supplementary Tables S1, S2) (35, 36). Isolates showing a non-wildtype pattern are referred as ‘resistant’ throughout this paper. Isolates showing resistance to at least three different antimicrobial classes are referred as ‘multidrug resistant’. Isolates showing a specific pattern as defined by EFSA (37) using the EUVSEC2 plate were referred as ESBL-producing *E. coli*, AmpC-producing *E.coli* or ESBL/AmpC-producing *E. coli*.

### 2.4. Statistical analyses

Resistance profiles of isolated commensal and ESBL/AmpC-producing *E. coli* in 2020 and 2022 were determined at farm level (combining observed ESBL/AmpC presence or class-specific resistance results from all isolates from the respective farm). The AMR data from both periods were used separately for analysis because the first sampling was done in the summer/fall of 2020 and not at the end of the year. Furthermore, it should be mentioned that the resistance data of the obtained isolates from the two paired boot swabs were combined in the group of cows. A farm was categorized as “not resistant” for a certain antibiotic class if all collected isolates of the three age groups showed full susceptibility to the respective antibiotic class. If an isolate in any age group showed resistance to the respective antibiotic class, the farm was categorized as “resistant” for this class. Furthermore, it should be noted that sulfonamides and trimethoprim were considered as belonging to the same class. This was also the case for nalidixic acid and ciprofloxacin, grouped in the class quinolones, as well as tetracycline and tigecycline, grouped in the class tetracyclines. A farm was classified as multidrug resistant if resistance was present to at least three antibiotic classes in at least one of the isolates in the tested age groups. Linkage between the presence of ESBL *E. coli*, multidrug resistance, as well as resistances toward the most frequently used antibiotic classes (i.e., cephalosporins, penicillins, quinolones, sulfonamides, and tetracyclines) each as a function of AMU were analyzed in separate linear binomial models. Aminoglycosides, amphenicols, and macrolides were not analyzed because they were applied by too few farms for the models to fit properly.

The variable indicating AMU was hereby either nDDDvet/cow/year in 2020 of the respective antibiotic class or combined classes, nDCD_vet_/cow/year in 2020 of the respective antibiotic class or combined classes, classified nDCD_vet_, or classified nDDD_vet_ (classification according to tertiles of the respective antibiotic class or combined classes in 2020), so that for each dependent variable four separate models were calculated, respectively. All binomial models were checked for overdispersion and showed no signs for serious overdispersion.

All statistical analyses were performed in R 4.2.2.^1^

## 3. Results

### 3.1. Antimicrobial use

**Figure 2:**
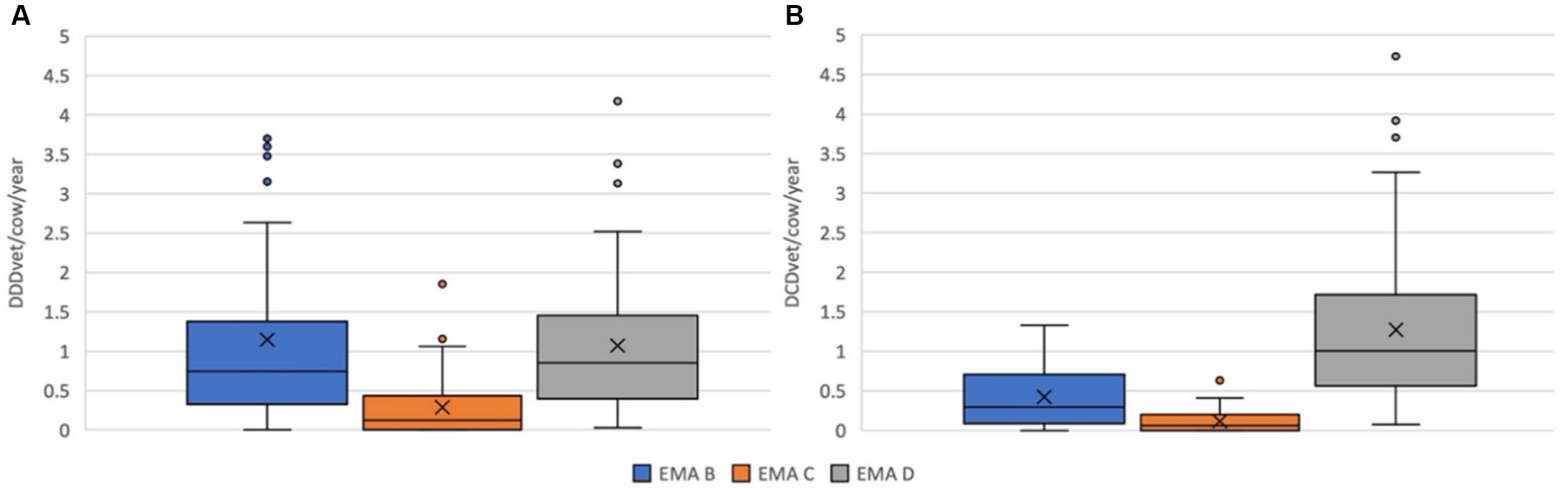
Antimicrobial use in **(A)** nDDDvet/cow/year and **(B)** nDCDvet/cow/year of the study farms by EMA categories. X—mean; horizontal line—median; box—range between 1st and 3rd quartile; dots—outliers.

**Figure 3:**
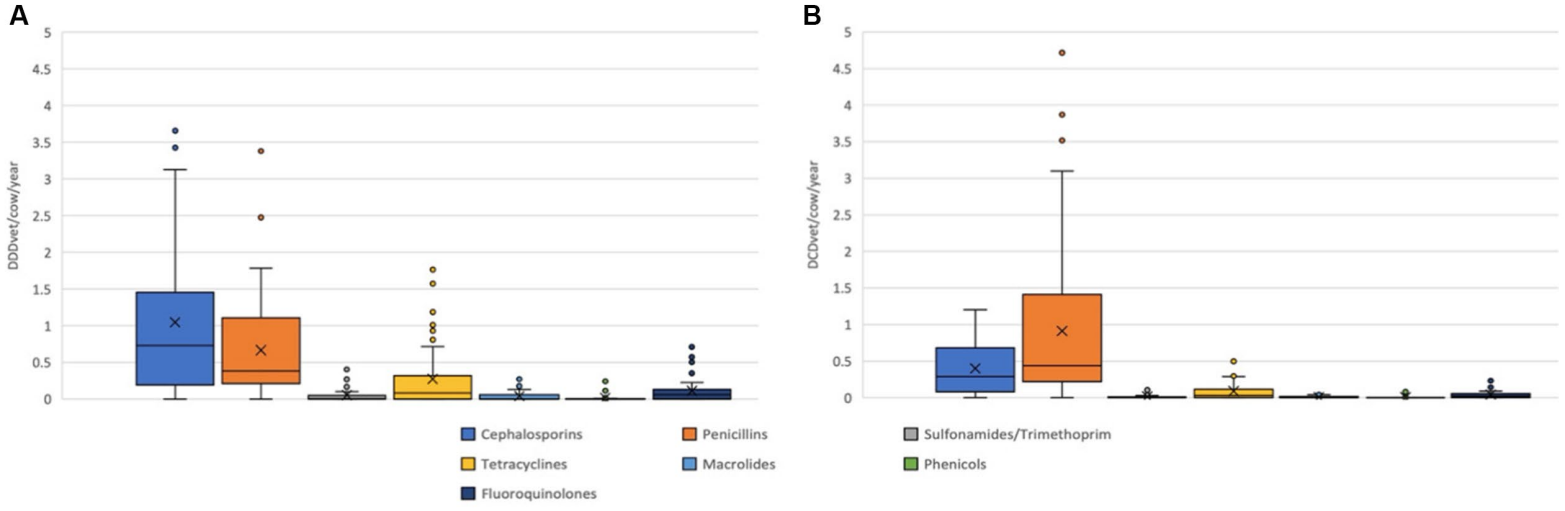
Antimicrobial use in **(A)** nDDDvet/cow/year and **(B)** nDCDvet/cow/year of the study farms by antimicrobial classes. X—mean; horizontal line—median; box—range between 1st and 3rd quartile; dots—outliers.

The nDDD_vet_/cow/year and farm for all antibiotics (excluding dry cow therapy) ranged from a minimum of 0.028 to a maximum of 6.910 (mean 2.504; median 2.580). The calculated nDCD_vet_/cow/year for all antibiotics (including dry cow therapy) varied from 0.407 to 4.730 (mean 1.812; median 1.571) per farm. Figures 2A,B show the distribution of nDDD_vet_/cow/year and nDCD_vet_/cow/year of the study farms by EMA categories (38). No EMA Category A antibiotics were used as these are not licensed for use in food-producing animals in the European Union. The distribution of nDDD_vet_/cow/year and nDCD_vet_/cow/year of the study farms for individual drug classes are shown on Figures 3A,B. Cephalosporins (mean 1.049; median 0.732 DDD_vet_/cow/year) and penicillins (mean 0.667; median 0.383 DDD_vet_/cow/year) were the most frequently used antibiotics on these farms, followed by tetracyclines (mean 0.275; median 0.084 DDD_vet_/cow/year). Only a very small amount of aminoglycosides and no polymyxins were used during the study period. A total of 142 oxytetracycline sprays were dispensed over the one-year period included here.

### 3.2. Bacteriological results

In 2020, a total of 603, and in spring 2022, a total of 587 fecal samples were collected from the 51 study farms. In 2020, samples from pre-weaned calves and weaned calves were collected from 50/51 farms each. In 2022, pre-weaned calves were present on 47/51 farms and weaned calves on 49/51 farms. After pooling of calf samples and sample processing in the laboratory, bacteriological results from 202 samples could be evaluated in 2020 and from 201 samples in 2022.

#### 3.2.1. Isolation and antimicrobial resistance of commensal *Escherichia coli*

In 2020, commensal *E. coli* could be isolated from 198 of the 202 (98.0%) fecal samples. The highest isolation rate for *E. coli* was obtained for weaned calves (100.0%, 50/50 of the pooled samples), whereas this was slightly lower for boot swabs from the cowshed (99.0%, 101/102 samples), and for pooled samples from pre-weaned calves (94.0%, 47/50 samples).

Of the 198 *E. coli* isolated in 2020, 53 isolates (26.8%) were resistant to at least one antibiotic class. Furthermore 35 (17.7%) isolates were classified as multidrug resistant (resistant against three or more antibiotic classes). By age group, the highest rate of commensal *E. coli* with at least one resistance was identified in pre-weaned calves (51.1%; 24/47), followed by 30.0% in weaned calves (15/50) and 13.9% in cows (14/101) (Table 1).

**Table 1:** Occurrence of at least one resistance in commensal *E. coli* in 2020 and 2022.

	*N* (%) of isolates with at least one resistance		*N* (%) of isolates with at least three resistances	
	2020	2022	2020	2022
Pre-weaned calves	24/47 (51.1%)	29/47 (61.7%)	19/47 (40.4%)	21/47 (44.7%)
Weaned calves	15/50 (30.0%)	16/49 (32.7%)	8/50 (16%)	6/49 (12.2%)
Cows	14/101 (13.9%)	19/94 (20.2%)	6/101 (5.9%)	6/94 (6.4%)
Number of farms*	33/51 (64.7%)*	34/51 (66.7%)*	24/51 (47.1%)**	21/51 (41.2%)**

* Total number of farms with at least one resistance, not number of samples.

** Total number of farms with MDR, not number of isolates.

**Figure 4:**
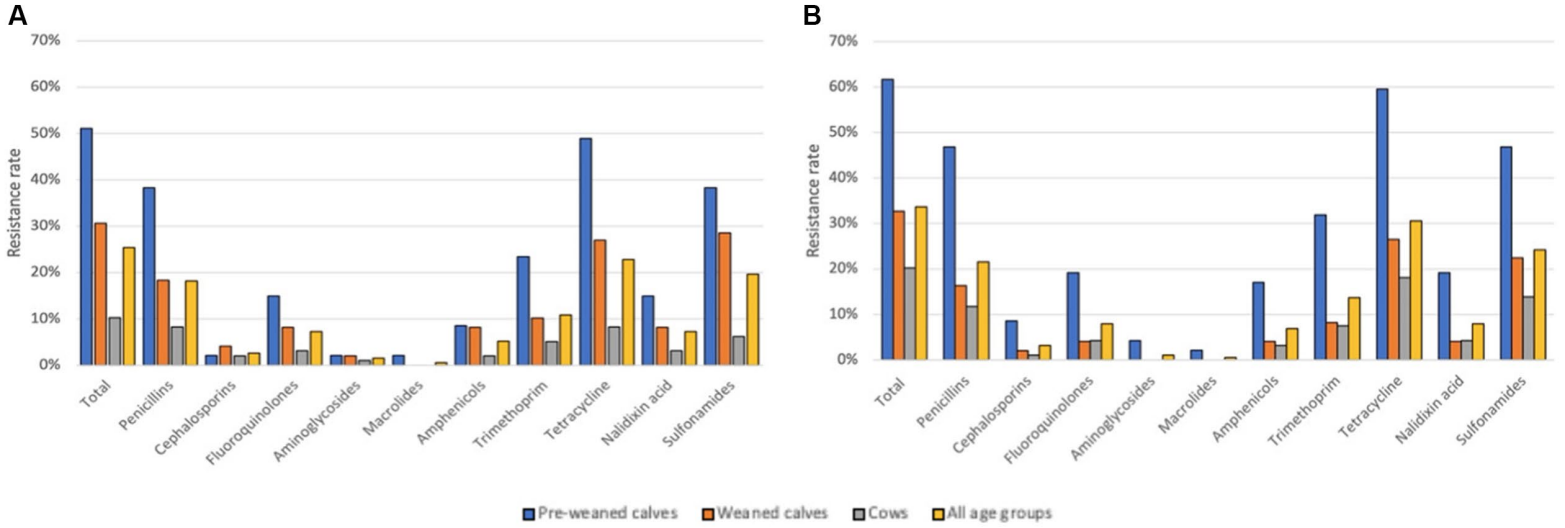
Resistance rates of commensal E. coli per antibiotic class in **(A)** 2020 and **(B)** 2022. Antibiotic classes represent following tested substances: Total-Isolates with at least one antibiotic resistance; Penicillin-Ampicillin; Cephalosporins- Cefotaxime, Ceftazidime; Quinolones-Ciprofloxacin, Nalidixic acid; Aminoglycosides-Gentamicin, Amikacin; Macrolides-Azithromycin; Phenicols-Chloramphenicol; Tetracyclines-Tetracycline, Tigecycline; Sulfonamides- Sulfamethoxazol.

All 198 commensal *E. coli* isolates were sensitive to polypeptides, carbapenems and tigecyclines. The most frequent resistance was determined to tetracyclines with 23.2% (46/198), sulfonamides with 20.2% (40/198) and penicillins with 19.2% (38/198) (Figure 4A). At least one resistant isolate was detected on 64.7% (33/51) of farms, while multidrug resistance was found on 47.1% (24/51) of farms, respectively (Table 1).

In 2022, from 190 out of 201 (94.5%) fecal samples an *E. coli* isolate could be collected for further analysis. Commensal *E. coli* could be isolated in 92.2% of samples from cows (94/102), 95.9% from pre-weaned calves (47/49) and 98% from weaned calves (49/50). A higher overall level of antimicrobial resistance was determined in 2022. Of the 190 isolates, 64 (33.7%) displayed resistance to one or more classes of antibiotics and 38 (20.0%) isolates were multidrug resistant.

Resistance to at least one antibiotic class was again most frequently determined among the pre-weaned calves (61.7%; 29/47 samples). In the group of weaned calves, 32.7% (16/49) of isolates and 20.2% (19/94) of cow samples contained resistant *E. coli* (Table 1). As in 2020, none of the samples were resistant to polypeptides, carbapenems and tigecyclines. Again, the highest rates of resistance among commensal *E. coli* were determined to tetracyclines (30.5%, 58/190), sulfonamides (24.2%, 46/190), and penicillins (21.6%, 41/190) (Figure 4B).

In 2022, *E. coli* with at least resistance to one antibiotic class could be isolated on 34 (66.7%) of the 51 farms. Multidrug resistant *E. coli* could be identified on 21 (41.2%) farms (Table 1).

#### 3.2.2. Detection of ESBL/AmpC-producing *Escherichia coli*

**Figure 5:**
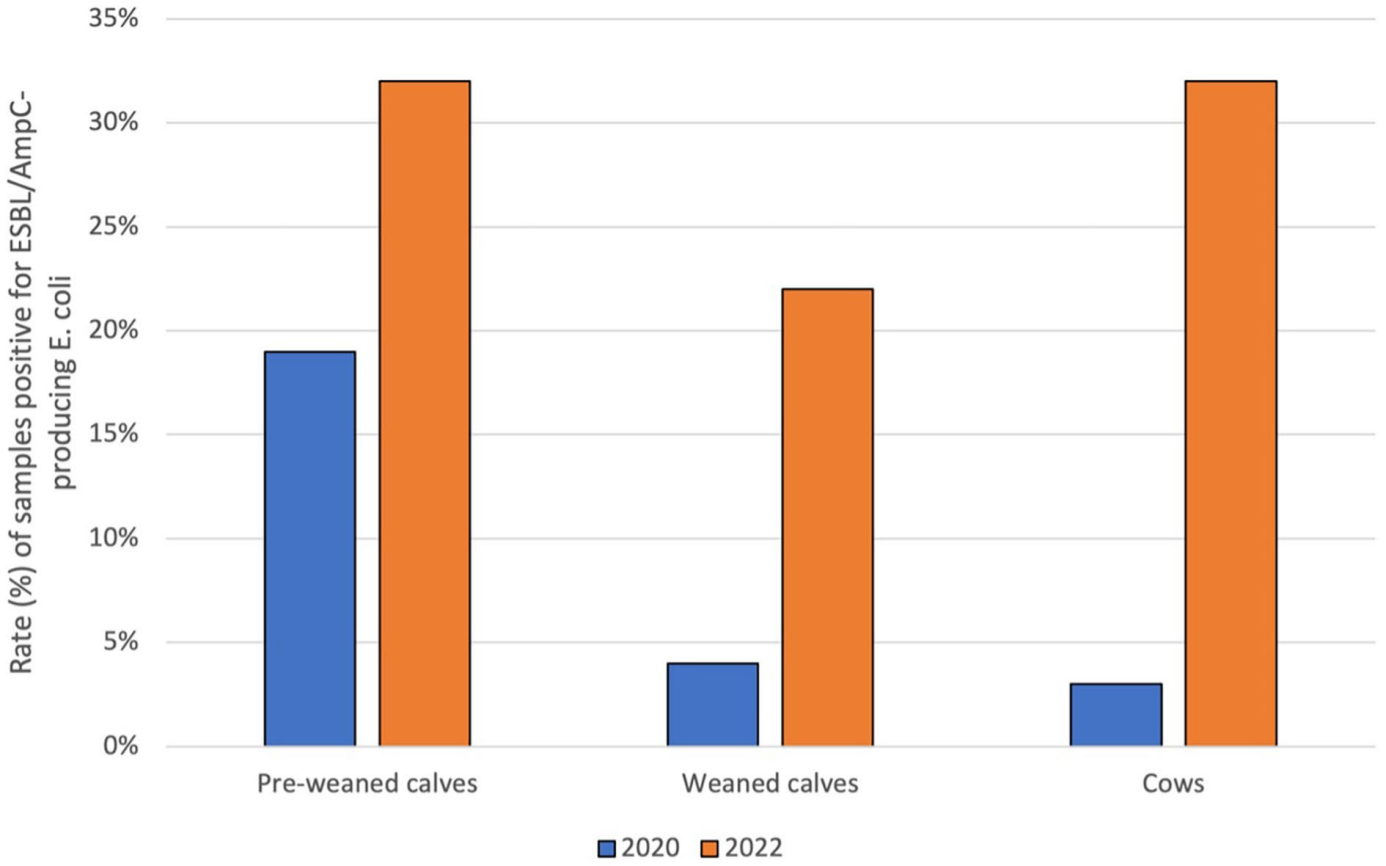
Comparison of the occurrence of ESBL/AmpC-producing *E. coli* in the different age groups at the two sampling periods.

In 2020, using a selective detection method to identify ESBL/AmpC-producing *E. coli*, 37 of 198 samples, where any *E. coli* could be isolated, were positive. These suspicious *E. coli* isolates were further tested to confirm ESBL/AmpC-production. Fourteen out of 198 tested samples were confirmed to be positive for ESBL-producing *E. coli* (7.1%) and one (0.5%) for AmpC-producing *E. coli*. By age group, ESBL-producing *E. coli* were isolated from 3/101 cow samples (3.0%), 9/47 pre-weaned calf samples (19.2%) and 2/50 weaned calf samples (4.0%) (Figure 5). In addition, the AmpC-producing *E. coli* isolate was identified in the rectal swabs from weaned calves. The occurrence of ESBL/AmpC-producing *E. coli* was limited to 11 (21.6%) farms in 2020.

In 2022, from a total of 190 samples with detection of any *E. coli* isolate, 58 suspicious isolates were identified using the selective method, of which 41 isolates (21.6%) were confirmed as ESBL-producing *E. coli*, ten as AmpC-producing *E. coli* (5.3%) and five as ESBL and AmpC-producing *E. coli* (2.6%). By age group, the detection rate of ESBL/AmpC-producing *E. coli* was 31.9% (30/94) in cow samples, 22.4% (11/49) in weaned calves and 31.9% (15/47) in pre-weaned calves (Figure 5). In 2022, ESBL/AmpC-producing *E. coli* were present on 25/51 farms (49.0%).

All ESBL/AmpC *E. coli* isolates in 2020 and 2022 were sensitive to polypeptides, carbapenems, and tigecycline. Of the 11 farms that tested positive for ESBL/AmpC-producing *E. coli* in 2020, ten were again positive in 2022.

### 3.3. Link between AMU and AMR

#### 3.3.1. Relationship between AMU and AMR in commensal *Escherichia coli* in 2020

In commensal *E. coli*, a trend toward a link was found between overall AMU (measured in the number of Defined Daily Dose (nDDD_vet_)/cow/year) and multiple resistances (≥ 3 antibiotic classes, not necessarily in the same isolate, but in the same age group; *p* = 0.067; Table 2).

**Table 2:** Link between antibiotic use (AMU) measured in nDDD_vet_/cow/year (numeric data or classified in tertiles) in 2020 and the presence of antibiotic resistance (AMR) to different antibiotic classes among commensal *E. coli* isolated from 51 farms in 2020.

AMR in 2020	Total AMU in 2020 (nDDD_vet_/cow/year)	Numeric AMU data				Classified AMU data			
		Est.	SE	*z*	*p*	Est.	SE	*z*	*p*
Multi-drug	All AB	0.39	0.21	1.83	0.067	0.99	0.72	1.38	0.168
Cephalosporins	Cephalosporins 3rd and 4th Gen.	0.37	0.37	1.00	0.317	0.47	0.99	0.48	0.630
Cephalosporins	All cephalosporins	0.13	0.39	0.34	0.734	−0.84	0.95	−0.88	0.376
Cephalosporins	All cephalosporins and all penicillins	0.17	0.35	0.50	0.620	0.00	1.06	0.00	1.000
Penicillins	All penicillins	0.55	0.47	1.17	0.244	1.48	0.74	2.01	0.044
Penicillin	All cephalosporins and all penicillins	0.29	0.25	1.18	0.238	1.21	0.72	1.69	0.091
Quinolones	Fluoroquinolones	2.44	1.89	1.29	0.197	2.17	1.15	1.89	0.059
Sulfonamides	Sulfonamide and trimethoprim	8.29	5.35	1.55	0.122	1.98	0.77	2.59	0.010
Tetracyclines	Tetracyclines	1.62	1.08	1.50	0.134	1.54	0.75	2.03	0.042

Antibiotic classes in AMR represent following tested substances: Penicillin-Ampicillin; Cephalosporins- Cefotaxime, Ceftazidime; Quinolones-Ciprofloxacin, Nalidixic acid; Tetracyclines-Tetracycline, Tigecycline; Sulfonamide- Sulfamethoxazol. For classified AMU data results are shown for the comparison of the two extreme conditions (i.e., low vs. high AMU; low vs. intermediate AMU not shown). Est, estimate; SE, standard error.

When AMU (measured in nDDD_vet_/cow/year) was divided into low, intermediate and high use (tertiles, “classified AMU data”), a significant association was identified between the high and low use of penicillins and resistance to ampicillin (*p* = 0.044; Table 2) as well as a trend toward a link between the total use of penicillins and cephalosporins and resistance to ampicillin (*p* = 0.091; Table 2). A statistically significant link between AMU and AMR was further determined for sulfonamides (*p* = 0.010) and tetracyclines (*p* = 0.042), and a strong trend for such a link was also found for fluoroquinolone use and quinolone resistance (all when classified into tertiles; *p* = 0.059; Table 2) among commensal *E. coli*. Results comparable to the latter three were also found when nDCD_vet_/cow/year divided into tertiles was used in the analysis for each antibiotic class namely sulfonamides: p = 0.010; tetracyclines: *p* = 0.084; quinolones: *p* = 0.059 (Table 3).

**Table 3:** Link between antibiotic use (AMU) measured in nDCD_vet_/cow/year (numeric data or classified in tertiles) in 2020 and the presence of antibiotic resistance (AMR) to different antibiotic classes among commensal *E. coli* isolated from 51 farms in 2020.

AMR in 2020	Total AMU in 2020 (nDCD_vet_/cow/year)	Numeric AMU data				Classified AMU data			
		Est.	SE	*z*	*p*	Est.	SE	*z*	*p*
Multi-drug	All AB	0.20	0.28	0.71	0.477	0.25	0.71	0.35	0.724
Cephalosporins	Cephalosporins 3rd and 4th Gen.	0.98	1.19	0.82	0.411	0.00	1.06	0.00	1.000
Cephalosporins	All cephalosporins	1.06	1.15	0.92	0.358	0.00	0.90	0.00	1.000
Cephalosporins	All cephalosporins and all penicillins	−0.23	0.45	−0.52	0.603	0.00	1.06	0.00	1.000
Penicillins	All penicillins	0.06	0.27	0.23	0.818	−0.49	0.70	−0.70	0.487
Penicillin	All cephalosporins and all penicillins	0.11	0.25	0.42	0.674	0.00	0.70	0.00	1.000
Quinolones	Fluoroquinolones	5.89	4.77	1.24	0.216	2.17	1.15	1.89	0.059
Sulfonamides	Sulfonamide and trimethoprim	23.22	15.26	1.52	0.128	1.98	0.77	2.59	0.010
Tetracyclines	Tetracyclines	3.58	2.89	1.24	0.216	1.30	0.75	1.73	0.084

Antibiotic classes in AMR represent following tested substances: Penicillin-Ampicillin; Cephalosporins- Cefotaxime, Ceftazidime; Quinolones-Ciprofloxacin, Nalidixic acid; Tetracyclines-Tetracycline, Tigecycline; Sulfonamide-Sulfamethoxazol. For classified AMU data results are shown for the comparison of the two extreme conditions (i.e., low vs. high AMU; low vs. intermediate AMU not shown). Est, estimate; SE, standard error.

#### 3.3.2. Relationship between AMU and AMR in commensal *Escherichia coli* in 2022

AMU data from 2020 were also compared with AMR determined in commensal *E. coli* from all farms in 2022. With respect to total AMU measured in nDDD_vet_/cow/year per antibiotic class, only fluoroquinolone use was determined to have a statistically significant association to resistance in commensal *E. coli* to quinolones (*p* = 0.023; Table 4). Although not statistically significant, a trend toward significance was also determined between penicillin use and resistance (*p* = 0.067; Table 4). Similar results were found when AMU data were classified into tertiles (penicillins: *p* = 0.091; quinolones: *p* = 0.065; Table 4).

**Table 4:** Link between antibiotic use (AMU) measured in nDDDvet/cow/year (numeric data or classified in tertiles) in 2020 and the presence of antibiotic resistance (AMR) to different antibiotic classes among commensal *E. coli* isolated from 51 farms in 2022.

AMR in 2022	Total AMU in 2020 (nDDD_vet_/cow/year)	Numeric AMU data				Classified AMU data			
		Est.	SE	*z*	*p*	Est.	SE	*z*	*p*
Multi-drug	All AB	0.05	0.18	0.27	0.784	−0.24	0.69	−0.34	0.732
Cephalosporins	Cephalosporins 3rd and 4th Gen.	0.11	0.39	0.28	0.781	−0.84	0.95	−0.88	0.376
Cephalosporins	All cephalosporins	−0.82	0.68	−1.20	0.230	−1.23	1.21	−1.02	0.309
Cephalosporins	All cephalosporins and all penicillins	−0.72	0.51	−1.42	0.157	−17.55	2.6	−0.01	0.995
Penicillins	All penicillins	0.95	0.52	1.83	0.067	1.21	0.72	1.69	0.091
Penicillin	All cephalosporins and all penicillins	−0.12	0.24	−0.53	0.598	−0.24	0.69	−0.34	0.732
Quinolones	Fluoroquinolones	4.76	2.10	2.27	0.023	1.66	0.90	1.84	0.065
Sulfonamides	Sulfonamide and trimethoprim	0.74	2.64	0.28	0.780	−0.09	0.65	−0.13	0.896
Tetracyclines	Tetracyclines	0.49	0.82	0.60	0.550	0.27	0.74	0.37	0.714

Antibiotic classes in AMR represent following tested substances: Penicillin-Ampicillin; Cephalosporins- Cefotaxime, Ceftazidime; Quinolones-Ciprofloxacin, Nalidixic acid; Tetracyclines- Tetracycline, Tigecycline; Sulfonamide- Sulfamethoxazol. For classified AMU data results are shown for the comparison of the two extreme conditions (i.e., low vs. high AMU; low vs. intermediate AMU not shown). Est, estimate; SE, standard error.

In 2022, a statistically significant negative association was determined with respect to total nDCD_vet_/cow/year for penicillin and cephalosporin use and the presence of resistance to cephalosporins in commensal *E. coli* (*p* = 0.042; Table 5). Fluoroquinolone use, measured in DCD_vet_/cow/year showed a statistically significant positive association with quinolone resistance in commensal *E. coli* (*p* = 0.021; Table 5). This effect also showed up as a trend when AMU was classified into tertiles (*p* = 0.065; Table 5).

**Table 5:** Link between antibiotic use (AMU) measured in nDCDvet/cow/year (numeric data or classified in tertiles) in 2020 and the presence of antibiotic resistance (AMR) to different antibiotic classes among commensal *E. coli* isolated from 51 farms in 2022.

AMR in 2022	Total AMU in 2020 (nDCD_vet_/cow/year)	Numeric AMU data				Classified AMU data			
		Est.	SE	*z*	*p*	Est.	SE	*z*	*p*
Multi-drug	All AB	0.03	0.26	0.11	0.915	−0.72	0.70	−1.03	0.303
Cephalosporins	Cephalosporins 3rd and 4th Gen.	1.43	1.19	1.20	0.229	0.47	0.99	0.48	0.630
Cephalosporins	All cephalosporins	−1.74	1.58	−1.11	0.269	−17.55	2.6	−0.01	0.995
Cephalosporins	All cephalosporins and all penicillins	−3.14	1.55	−2.03	0.042	−18.39	2.6	−0.01	0.994
Penicillins	All penicillins	−0.07	0.26	−0.28	0.778	−0.96	0.71	−1.36	0.174
Penicillin	All cephalosporins and all penicillins	−0.18	0.25	−0.74	0.462	−1.48	0.74	−2.01	0.044
Quinolones	Fluoroquinolones	12.77	5.54	2.30	0.021	1.66	0.90	1.84	0.065
Sulfonamides	Sulfonamide and trimethoprim	0.84	8.93	0.09	0.925	−0.09	0.65	−0.13	0.896
Tetracyclines	Tetracyclines	1.38	2.54	0.54	0.588	1.18	0.80	1.47	0.141

Antibiotic classes in AMR represent following tested substances: Penicillin-Ampicillin; Cephalosporins- Cefotaxime, Ceftazidime; Quinolones-Ciprofloxacin, Nalidixic acid; Tetracyclines-Tetracycline, Tigecycline; Sulfonamide- Sulfamethoxazol. For classified AMU data results are shown for the comparison of the two extreme conditions (i.e., low vs. high AMU; low vs. intermediate AMU not shown). Est, estimate; SE, standard error.

#### 3.3.3. Relationship between AMU and ESBL/AmpC-producing *Escherichia coli*

Analysis at farm level showed hardly any link between the level of use of penicillins and cephalosporins and the detection of ESBL/AmpC-producing *E. coli*. Only when total usage of penicillins and cephalosporins during lactation and dry-cow therapy in 2020 are combined (i.e., measured as nDCD_vet_/cow/year) could a significant negative association be observed for the detection of ESBL/AmpC-producing *E. coli* in 2022, both for numeric data (*p* = 0.029) and when classified into tertiles (*p* = 0.044; Table 6). It is particularly important to note, however, that these analyses were carried out on a small sample size of 11 farms in 2020 and 25 farms in 2022, and as such, no definite conclusions can be drawn.

**Table 6:** Link between the application of penicillins and cephalosporins (measured as nDDD_vet_/cow/year or nDCD_vet_/cow/year; *numeric data or classified in tertiles*) in the year 2020 and the presence of ESBL/AmpC-producing *E. coli* in the two study periods.

ESBL/AmpC- *E. coli*-presence in study period (farms positive)	Use of cephalosporins and penicillins	Numeric AMU data				Classified AMU data			
		Est.	SE	*z*	*p*	Est.	SE	*z*	*p*
2020 (*n* = 11)	nDCD_vet_/cow/year	−0.76	0.50	−1.54	0.124	−1.41	0.91	−1.55	0.121
	nDDD_vet_/cow/year	0.15	0.28	0.55	0.582	0.00	0.90	0.00	1.000
2022 (*n* = 25)	nDCD_vet_/cow/year	−0.71	0.32	−2.18	0.029	−1.48	0.74	−2.01	0.044
	nDDD_vet_/cow/year	0.08	0.24	0.34	0.736	0.00	0.69	0.00	1.000

For classified AMU data results are shown for the comparison of the two extreme conditions (i.e., low vs. high AMU; low vs. intermediate AMU not shown). Est, estimate; SE, standard error.

## 4. Discussion

The aim of this research was to investigate the prevalence of ESBL/AmpC-producing *E. coli* and the resistance pattern of commensal *E. coli* in different age groups on dairy cattle farms in Austria. In addition, the relationship between antimicrobial resistance and antibiotic use on these farms was investigated.

The present study recorded a mean AMU value of 2.504 DDDvet/cow/year (median 2.580; minimum 0.028 to a maximum of 6.910 DDD_vet_/cow/year). A previous study from Austria, using a comparable approach, calculated values ranging from a mean of 0.29 DDDvet/cow/year (median 0.31) in a group of farms classed as “low antibiotic users” to a mean of 4.25 DDDvet/cow/year (median 3.82) among “high antibiotic users” (39). By comparison, in 2007, a study conducted in the United States found that conventional dairy herds had an mean AMU of 5.43 DDD/cow/year (40). A study from Belgium reported that adult dairy cattle had a higher mean antimicrobial treatment incidence of 20.78 defined daily doses animal (DDDA) per 1,000 cow-days (approximately 7.58 DDD/cow/year) (41). Similarly, a study conducted in the Netherlands from 2005 to 2012 analyzed data from 94 dairy farms and found that the mean DDDA was 5.86 per cow and year (42). In contrast, an analysis of national data on intramammary therapies in Irish dairy herds conducted in 2015 revealed a much lower mean AMU of 1.398 DDD_vet_/cow/year and 1.022 DCD_vet_/cow/year (43). The various metrics used to measure antibiotic use in different published studies make it challenging to make comparisons; however, the data suggest that the dairy farms participating in the present study have a relatively low level of antibiotic consumption. Smaller herd sizes, such as those included in this study, which allow for better individual animal observation and care, could be a potential reason for the lower antibiotic consumption observed (44). In addition, Austria has a relatively low level of agricultural intensification, a high proportion of organic farms (22%), and a high use of dual-purpose breeds (75% of the national herd) (45, 46) which might be less sensitive to bacteriological infections. Furthermore, it is likely that veterinarians and farmers applying prudent antibiotic use principles might have agreed to participate in the study. Nevertheless, the authors believe that the collection of veterinary prescription data over an entire calendar year provided an accurate account of AMU on the dairy farms in the study population in the year 2020. Dispensed antibiotic sprays were not included in the analysis of AMU, because the frequency and volume of antibiotics applied by aerosol spray is extremely difficult to quantify and individual applications are rarely documented (47). While tetracycline resistance was relatively common on the farms investigated here, the authors do not believe that oxytetracycline sprays (one treatment of which has previously been estimated to use approximately 3 mL of product per 3 s spray (47), with the entire 150 mL spray can containing 390 mg of oxytetracycline hydrochloride), significantly impacted the likelihood of occurrence of AMR on farm, compared to the systemic use of tetracyclines. A number of other authors have found tetracycline resistance to be relatively common on dairy farms and their environment, even where tetracyclines are not frequently used (48–51).

It is important to note that cephalosporins were the most frequently used antibiotics on the participating dairy farms. This is a common finding and the frequent use of cephalosporins been reported elsewhere on dairy farms worldwide (31, 40, 41, 43). Nevertheless, third and fourth generation cephalosporins are classed by the European Union as Category B antibiotics, which are critically important to human medicine as part of the One Health concept. As such, their use in veterinary medicine should be reduced and the more restricted use of Category B antibiotics as set out in the new EU regulation (2019/6) on veterinary medicinal products (which was not in force at the time of this data collection) should ensure the more prudent use of these antibiotics in future.

In the initial sampling period conducted in 2020, 7.6% of the samples were found to be positive for ESBL/AmpC-producing *E. coli*. These isolates were obtained from 11 farms (21.6% of all farms), and the majority of ESBL/AmpC-producing *E. coli* were identified in pre-weaned calves (64.3% of all ESBL/AmpC isolates across all age groups, and 19.2% of all pre-weaned calf samples). In 2022, a higher percentage of ESBL/AmpC-producing *E. coli* was observed in the study population, with 29.5% of isolates classified as ESBL/AmpC-producing *E. coli*. These isolates were obtained from 25 farms (49.0% of all farms). Among the individual groups, the highest proportion of positive isolates was found in samples from pre-weaned calves and cows, each with almost 32%. Additionally, 22.5% of samples obtained from weaned calves were positive for ESBL/AmpC-producing *E. coli*. A previous study conducted in Austria in 2017 analyzed voided fecal samples from cowsheds, calf pens and youngstock housing areas, and found that 26% of dairy farms had ESBL/AmpC-producing *E. coli* present (39).

The prevalence of ESBL/AmpC-producing *E. coli* has been reported to be much higher in other countries, e.g., in a study carried out in Germany in 2011/2012, ESBL-producing *E. coli* could be isolated from fecal samples, boots swabs and dust samples on 86.7% of cattle (both beef and dairy) farms (52). Another study from Germany run in 2018/2019 examined fecal samples of calves and dams of 72 large dairy farms and found at least one positive sample for ESBL-producing *E. coli* on all farms (53). Furthermore, a very high prevalence of ESBL/AmpC-producing *E. coli* was found in fecal samples of calves, cows and the manure pit in a study performed in Canada, with 85% positivity on dairy farms (54). In a further study conducted in the Netherlands, a high prevalence of ESBL/AmpC-producing *E. coli* was detected in fecal samples of calves, youngstock and dairy cows on 59.6% of the participating dairy farms (55). A study from England and Wales determined a prevalence of 35% positive samples for ESBL-producing *E. coli* on dairy farms (19). In contrast, a much lower prevalence was found in a study from Japan with 5.2% in all age groups of dairy herds (56). However, it is important to note that the aforementioned studies all differ in a variety of aspects, such as the selection and size of the population (dairy farm or beef farm), the material sampled (slurry, rectal swabs), the number of samples taken per farm, and the selection or identification method of the ESBL/AmpC-producing *E. coli* in the laboratory. Although the results cannot be directly compared, it is important to note that all the studies that examined fecal samples from several age groups were able to demonstrate the same trend, namely that the youngest calves have the highest prevalence of ESBL/AmpC-producing *E. coli*.

In the context of routine European Union antimicrobial resistance monitoring, where the caecal content of slaughtered calves (<1 year) is investigated, a prevalence of 22.4% for presumptive ESBL and/or AmpC-producing *E. coli* was reported for samples collected in Austria in 2017, which is in line with the results presented here. In the same report, Germany was determined to have an ESBL and/or AmpC-producing *E. coli* prevalence of 67.7%, the Netherlands 37.7%, Italy 89.0% and the lowest was Denmark with 7.1% (37).

In the most recent report on the resistance monitoring published in 2021, no increase of ESBL/AmpC-producing *E. coli* prevalence was observed. The respective detection rates were lower (Germany, Netherlands, Denmark) or at the same level (Italy) as in 2017 (57). Due to the low number of calves produced for slaughter, Austria was not required to repeat the sampling of calves for the 2021 report, thus no updated information using the same study protocol is available.

The observed increase in the occurrence of ESBL/AmpC-producing *E. coli* between 2020 and 2022 in the present study could potentially be attributed to the use of different selective agar plates in the laboratory during these two periods. Additionally, research on resistant isolates in humans in the USA suggests that environmental temperature could impact the occurrence of ESBL-producing *E. coli* (58). Given that samples in the present study were collected in different seasons, environmental temperatures could potentially impact the occurrence or detection of ESBL-producing *E. coli.*

The most common resistances determined among commensal *E. coli* isolates were to tetracyclines (23% in 2020 and 31% in 2022), sulfonamides (20% in 2020 and 24% in 2022) and penicillins (19% in 2020 and 22% in 2022). In addition, there was a notable increase in the presence of multidrug resistance among the isolates, with rates of 18% in 2020 and 20% in 2022. Nevertheless, in the current study, even in 2022, 66% of the *E. coli* isolates were fully susceptible to antibiotics. In the JIACRA III report, *E. coli* isolates from bovines in Austria under 1 year of age, collected for cecal content during slaughter, were found to have an overall complete susceptibility of 73.5%. Other countries, such as Germany and the Netherlands, had lower levels of complete susceptibility (53.3 and 49.5%). Countries in Northern Europe, such as Denmark and Norway, performed best (up to almost 95% complete susceptibility), while Italy had the highest resistance rate, with a complete susceptibility rate of only 19.4%. It should be noted, however, that in the JIACRA III report isolates from broilers, turkeys, pigs and veal calves are considered in a combined figure, rather than just bovine animals of all ages as the report aims to provide an analysis of the overarching trends in AMR and AMU (59).

In the present study, the highest level of antibiotic resistance in both commensal and ESBL/AmpC-producing *E. coli* was found in pre-weaned calves (<6 weeks of age), followed by weaned calves (>6 weeks of age) and then cows. Many other authors have reported similar findings, namely that pre-weaned calves have the highest levels of antibiotic resistance and that these rates decrease with age (22, 60–63). For this reason, pre-weaned calves could act as sentinel animals for the presence of AMR in the herd. In many studies, the main reason for the high prevalence of AMR, apart from AMU, is associated with poor hygiene and, in the case of pre-weaned calves, often with the feeding of waste milk containing antibiotic residues (64–68). A study from England in 2011 noted that ESBL-producing *E. coli* can be isolated in waste milk in addition to antibiotic residues (69). While the feeding of waste milk containing antibiotic residues to calves is permitted in Austria and this cannot be ruled out as a cause of higher AMR rates among this age group, recently, researchers from the Netherlands (where waste milk feeding is not permitted) similarly reported a high prevalence of ESBL/AmpC-producing *E. coli* among pre-weaned calves (33.7% aged 0 to 20 days old). The authors of the Dutch study suggested that the gut of calves in the first days of life is highly susceptible to colonization with these resistant bacteria, which the calves may acquire from their environment and that selection of resistant bacteria in the gut due to antimicrobial treatment of the calves can also result in colonization of the gut (70).

Antibiotic treatment of calves themselves is not likely to be the reason for the high prevalence of AMR and ESBL-producing *E. coli* in this age-class in this study, as recorded calf treatments made up an extremely small proportion of the reported AMU (<5%). In other countries (such as the Netherlands and Switzerland), veal calves are frequently treated with antibiotics (71, 72), but such an intensive market for calves does not exist in Austria and the dual-purpose *Fleckvieh* calves are generally reared to adulthood and it is important to note that the present study only included primarily dairy farms. It is more likely that pre-weaned calves received waste milk containing antibiotic residues, however this information was not recorded for each calf from which fecal samples were taken in this study. Nevertheless, many other authors have also noted that pre-weaned calves are much more likely to harbor AMR bacteria in their intestines (26, 73, 74). Weber et al. suggest that cows with higher fitness levels may have lower colonization rates of ESBL/AmpC-producing *E. coli*, which could potentially reduce the risk of infection for calves during birth (53). In the present study, it was only possible to analyze the AMU data from the entire herd, and not from individual calves. Although documentation of calf treatment is required, it is often not as precise as that for the treatment of adult cows. This is due to the fact that calves often do not have ear tags yet and may be treated as a group within one identification number. Therefore, it was not possible to determine which antibiotics, if any, the calves had received, nor whether the calves from which fecal samples were collected had previously been treated with antibiotics.

The analyses of the relationship between AMU and AMR of 2020 showed that there was a trend toward a link between the overall use of antibiotics (calculated as nDDD_vet_/cow/year) and the presence of multidrug resistance among commensal *E. coli*. A statistically significant association was found between the use of penicillins and resistance to ampicillin and a trend toward a link between the total use of penicillins and cephalosporins and resistance to ampicillin (both calculated as nDDD_vet_/cow/year; divided into tertiles). A study conducted in China in 2021, investigating the impact of therapeutic administration of cephalosporin antibiotics on the bacterial community and antibiotic resistance patterns in milk, reported similar findings. It revealed that the increased usage of cephalosporins was associated with an elevation in the presence of beta-lactam resistance genes (75). A study conducted by Pereira and colleagues, which investigated the antibiotic usage and the occurrence of resistances in calf feces, also concluded that the administration of cephalosporins resulted in an increased prevalence of multidrug resistance (73).

Here a statistically significant association between AMU and AMR was also found for sulfonamides and tetracyclines (measured either as nDDD_vet_/cow/year and nDCD_vet_/cow/year; when divided into tertiles). In the analyses of the relationship between AMU und AMR of 2022, a significant link between the AMU of fluoroquinolones (calculated as nDDD_vet_/cow/year) and AMR against quinolones could be identified and a trend toward a link of AMU and AMR of penicillins could be demonstrated.

A study in Germany demonstrated that farms with no AMU had a significantly lower number of ESBL-producing *E. coli* detected than farms with typical antibiotic use (52). Studies from the Netherlands (21, 76) have also demonstrated that higher 3rd and 4th generation cephalosporin use increased the risk of ESBL-producing *E. coli* being present on farm. This phenomenon has also been described in other food-producing animals, such as turkey flocks in Canada (77). According to the JIACRA III report, there appears to be a correlation between AMU and AMR at national level in the EU. This is particularly notable in countries with extremely high and extremely low AMU. For example, Sweden and Finland had a low mean antibiotic usage (AMU) of 11.9 and 20.2 milligrams per Population Correction Unit (PCU) respectively, with a mean complete antibiotic susceptibility of *E. coli* of 70.2 and 76.3% for the period 2014–2018. In contrast, Greece and Italy had a much higher mean AMU of 76.4 and 273.4 milligrams per PCU for the same period, with a much lower complete antibiotic susceptibility of *E. coli* of 6.5 and 11.4%, respectively. While such relationships between AMU and AMR at farm or herd level are difficult to confirm, a significant association has been reported between the total national antibiotic usage (AMU) in food-producing animals and the percentage of *E. coli* strains that are resistant to third-generation cephalosporins in all participating EU countries included in the JIACRA III report, with a value of p less than 0.001 (59).

A limitation of this study was the selection of the participating farms and veterinarians as a convenience sample. In the four federal states included here, veterinarians were invited to participate in the study and recruit farmers. Furthermore, the number of participating farms is not representative for the whole of Austria and data for the calculation of nDDD_vet_/cow/year and nDCD_vet_/cow/year for these farms were only available for the whole year of 2020.

Due to COVID-19 lockdowns and restrictions, it was not possible to collect fecal samples in the same season each time or in 2021. The development of antibiotic resistance in bacteria and their spread is a complex process that can involve various mechanisms, such as mutation, acquisition of resistance genes, and horizontal gene transfer. While some mechanisms can occur quickly, others can take longer to develop (78). As this was an observational study investigating AMR at herd, rather than animal level, it is important to look at the long-term patterns of antimicrobial use on farms to better understand the potential link with antimicrobial resistance. Short-term studies may provide valuable insights, but they may not be sufficient to draw valid conclusions about the relationship between antimicrobial use and resistance. Another important aspect that should not be ignored is that the sampling methodology and sample handling may have an influence on the identification of resistances (79). The use of different selective agar plates for the bacteriological investigation of the second sampling period was due to the unavailability of the plates used in the first sampling period. However, since the same researchers were responsible for the sample collection, dispatch, and evaluation in the national reference laboratory for antibiotic resistance in both years, this factor should not have impacted on the results.

## 5. Conclusion

This study assessed the level of antimicrobial resistance and occurrence of ESBL/AmpC-producing *E. coli* on 51 dairy farms in four federal states of Austria by collecting fecal samples twice and comparing them with the AMU over a previous one-year period. The most commonly determined resistances were to tetracyclines, sulfonamides, and penicillins and there was a moderate prevalence of ESBL/AmpC-producing *E. coli*. Among commensal *E. coli* isolated on farms in 2020, when antibiotic use was classified into tertiles of low, medium and high use, then AMU measured in nDDDvet/cow/year displayed a statistically significant link to AMR with respect to penicillin use and ampicillin resistance, as well as tetracycline use and sulfonamide/trimethoprim use and their respective resistances. Furthermore, a tendency toward a statistically significant association was identified between overall AMU in 2020 (by nDDD_vet_/cow/year) and multidrug resistances in commensal *E. coli* on farm. From samples collected in 2022, a statistically significant effect was determined between fluoroquinolone use (measured in both nDDD_vet_/cow/year and nDCD_vet_/cow/year) and resistance to quinolones in commensal *E. coli*.

Since the selection and spread of antimicrobial resistance is a complex and multifactorial process, it is important to take into account a variety of factors such as management practices, hygiene standards, environmental parameters, and longer periods of antimicrobial use in order to gain a better understanding of the underlying mechanisms. Therefore, further studies are needed to investigate these factors and their impact on the development of antimicrobial resistance in *E. coli* on dairy farms.

## Data availability statement

The datasets presented in this article are not readily available because the authors of the study do not have ownership of the antimicrobial use and production data used in the analysis. The data were provided by the veterinarians who treated the animals in the study, and by the ZuchtData EDV-Dienstleistungen GmbH who coordinate the national cattle production database, and are subject to a data privacy agreement that prohibits their publication. Requests to access the datasets should be directed to CF, clair.firth@vetmeduni.ac.at.

## Ethics statement

The animal study was reviewed and approved by institutional ethics and animal welfare committee in accordance with GSP guidelines and national legislation (ETK-34/02/2019). Written informed consent was obtained from the owners for the participation of their animals in this study.

## Author contributions

CF, WO, CE-D, and AK designed and developed the study protocol. TW and CF collected AMU data and samples on farm and wrote the manuscript. BW and SK-J developed the laboratory protocols and carried out the laboratory analyses. CF, TW, WO, KF, CE-D, SV, and AK analyzed the AMU and AMR data. SV carried out the statistical analysis. CF, WO, and AK supervised the project. All authors reviewed and revised the manuscript.

## Funding

This work was conducted within the COMET-Project D4Dairy (Digitalisation, Data integration, Detection and Decision support in Dairying, Project number: 872039) that is supported by BMK (Austrian Federal Ministry of Climate Action, Environment, Energy, Mobility, Innovation and Technology), BMDW (Austrian Federal Ministry of Digital and Economic Affairs) and the provinces of Lower Austria and Vienna in the framework of COMET-Competence Centers for Excellent Technologies. The COMET program is handled by the FFG (grant number 872039).

## Conflict of interest

CE-D is employed by ZuchtData EDV-Dienstleistungen GmbH; WO owns his own veterinary practice. All authors collaborated on this project as part of the D4Dairy research consortium (www.d4dairy.com), which was made up of both commercial and academic research institutions as required by the funding agency. The funders did not contribute to the study’s design, data collection, analysis or interpretation, manuscript writing, or decision to publish the results.

The remaining authors declare that the research was conducted in the absence of any commercial or financial relationships that could be construed as a potential conflict of interest.

## Publisher’s note

All claims expressed in this article are solely those of the authors and do not necessarily represent those of their affiliated organizations, or those of the publisher, the editors and the reviewers. Any product that may be evaluated in this article, or claim that may be made by its manufacturer, is not guaranteed or endorsed by the publisher.

## Supplementary material

The Supplementary material for this article can be found online at: https://www.frontiersin.org/articles/10.3389/fvets.2023.1225826/full#supplementary-material

## Footnotes

^1^ www.r-project.org

## References

[1] World Health Organization. WHO global strategy for containment of antimicrobial resistance, world health Organisatin. WHO glob Strateg contain Antimicrob resist. Geneva: World Health Organization, pp. 1–105. (2001).

[2] O’Neill J. *Tackling drug-resistant infections globally: Final report and recommendations. Review on antimicrobial resistance. Wellcome Trust and HM government*. (2016). Available at: https://amr-review.org/sites/default/files/160525_Finalpaper_withcover.pdf.

[3] Murray CJ Ikuta KS Sharara F Swetschinski L Robles Aguilar G Gray A. Global burden of bacterial antimicrobial resistance in 2019: a systematic analysis. Lancet. (2022) 399:629–55. doi: 10.1016/S0140-6736(21)02724-0, PMID: 35065702 PMC8841637

[4] Ventola CL. The antibiotic resistance crisis: part 1: causes and threats. P T. (2015) 40:277–83. 25859123 PMC4378521

[5] Garcia-Migura L Hendriksen RS Fraile L Aarestrup FM. Antimicrobial resistance of zoonotic and commensal bacteria in Europe: the missing link between consumption and resistance in veterinary medicine. Vet Microbiol. (2014) 170:1–9. doi: 10.1016/j.vetmic.2014.01.013, PMID: 24589430

[6] Ewers C Bethe A Semmler T Guenther S Wieler LH. Extended-spectrum β-lactamase-producing and AmpC-producing *Escherichia coli* from livestock and companion animals, and their putative impact on public health: A global perspective. Clin Microbiol Infect. (2012) 18:646–55. doi: 10.1111/j.1469-0691.2012.03850.x 22519858

[7] Dahms C Hübner N-O Kossow A Mellmann A Dittmann K Kramer A. Occurrence of ESBL-producing *Escherichia coli* in livestock and farm Workers in Mecklenburg-Western Pomerania, Germany. PLoS One. (2015) 10:e0143326. doi: 10.1371/journal.pone.0143326, PMID: 26606146 PMC4659621

[8] European Commission. Regulation (EC) no 1831/2003 of the European Parliament and of the council on additives for use in animal nutrition. Brussels: European Commission (2003).

[9] BMGF. *Tierarzneimittelkontrollgesetz—TAKG (control of veterinary medicinal products law)*. (2002) Available at: https://www.ris.bka.gv.at/GeltendeFassung.wxe?Abfrage=Bundesnormen&Gesetzesnummer=20001741%0ABondt (Accessed October 27, 2017).

[10] AVMA. *The veterinarian-client-patient relationship (VCPR)*. (2003). Available at: https://www.avma.org/resources-tools/pet-owners/petcare/veterinarian-client-patient-relationship-vcpr.

[11] BMGF. *Tiergesundheitsdienst–Verordnung 2009–TGD-VO 2009*. (2009). Available at: https://www.ris.bka.gv.at/GeltendeFassung.wxe?Abfrage=Bundesnormen&Gesetzesnummer=20006592.

[12] BMGF. *Veterinär-Antibiotika–MengenströmeVO*, pp. 1–7. (2014). Available at: https://www.ris.bka.gv.at/eli/bgbl/II/2014/83.

[13] Fuchs R Fuchs K. *Antibiotika-Vertriebsmengen in der Veterinärmedizin*. (2022). Available at: https://www.ages.at/tier/tierarzneimittel-hormone/antibiotika-vertriebsmengen-in-der-veterinaermedizin.

[14] Selbitz HJ Truyen U Valentin-Weigand P. *Tiermedizinische Mikrobiologie, Infektions- und Seuchenlehre*. Stuttgart: Georg Thieme Verlag. (2015). Available at: http://www.thieme-connect.de/products/ebooks/book/10.1055/b-003-127007.

[15] Theuretzbacher U. Β-Lactamasen. Pharm Unserer Zeit. (2006) 35:416–21. doi: 10.1002/pauz.200600187, PMID: 17009785

[16] Pitout JD Laupland KB. Extended-spectrum β-lactamase-producing Enterobacteriaceae: an emerging public-health concern. Lancet Infect Dis. (2008) 8:159–66. doi: 10.1016/S1473-3099(08)70041-0 18291338

[17] Perestrelo S Amaro A Brouwer MSM Clemente L Ribeiro Duarte AS Kaesbohrer A. Building an international one health strain level database to characterise the epidemiology of AMR threats: ESBL—AmpC Producing *E. coli* as an example–challenges and perspectives. Antibiotics. (2023) 12:552. doi: 10.3390/antibiotics12030552 36978419 PMC10044432

[18] Mughini-Gras L Dorado-García A Van Duijkeren E Van den Bunt G Dierikx CM Bonten MJM. Attributable sources of community-acquired carriage of *Escherichia coli* containing β-lactam antibiotic resistance genes: a population-based modelling study. Lancet Planet Health. (2019) 3:e357–69. doi: 10.1016/S2542-5196(19)30130-5, PMID: 31439317

[19] Snow LC Warner RG Cheney T Wearing H Stokes M Harris K. Risk factors associated with extended spectrum beta-lactamase *Escherichia coli* (CTX-M) on dairy farms in north West England and North Wales. Prev Vet Med. (2012) 106:225–34. doi: 10.1016/j.prevetmed.2012.03.009, PMID: 22552330

[20] Chantziaras I Boyen F Callens B Dewulf J. Correlation between veterinary antimicrobial use and antimicrobial resistance in food-producing animals: a report on seven countries. J Antimicrob Chemother. (2014) 69:827–34. doi: 10.1093/jac/dkt443 PMID: 24216767

[21] Gonggrijp MA Santman-Berends IMGA Heuvelink AE Buter GJ van Schaik G Hage JJ. Prevalence and risk factors for extended-spectrum β-lactamase- and AmpC-producing *Escherichia coli* in dairy farms. J Dairy Sci [Internet]. (2016) 99:9001–13. doi: 10.3168/jds.2016-11134, PMID: 27638264

[22] Gay E Bour M Cazeau G Jarrige N Martineau C Madec JY. Antimicrobial usages and antimicrobial resistance in commensal *Escherichia coli* from veal calves in France: evolution during the fattening process. Front Microbiol. (2019) 10:792. doi: 10.3389/fmicb.2019.00792, PMID: 31031738 PMC6473463

[23] OECD. *Antimicrobial resistance in the EU/EEA-A one health response*. (2022). Available at: https://www.oecd.org/health/Antimicrobial-Resistance-in-the-EU-EEA-A-One-Health-Response-March-2022.pdf.

[24] EMA/EFSA. EMA and EFSA joint scientific opinion on measures to reduce the need to use antimicrobial agents in animal husbandry in the European Union, and the resulting impacts on food safety (RONAFA). EFSA J. (2017) 15:e04666. doi: 10.2903/j.efsa.2017.4666, PMID: 32625259 PMC7010070

[25] Tragesser LA Wittum TE Funk JA Winokur PL Rajala-Schultz PJ. Association between ceftiofur use and isolation of *Escherichia coli* with reduced susceptibility to ceftriaxone from fecal samples of diary cows. Am J Vet Res. (2006) 67:1696–700. doi: 10.2460/ajvr.67.10.1696, PMID: 17014318

[26] Maynou G Bach A Terré M. Feeding of waste milk to Holstein calves affects antimicrobial resistance of Escherichia coli and *Pasteurella multocida* isolated from fecal and nasal swabs. J Dairy Sci. (2017) 100:2682–94. doi: 10.3168/jds.2016-11891, PMID: 28215892

[27] EMA. *Principles on assignment of defined daily dose for animals (DDDvet) and defined course dose for animals (DCDvet)*, p. 68. (2015). Available at: http://www.ema.europa.eu/docs/en_GB/document_library/Scientific_guideline/2015/06/WC500188890.pdf.

[28] EMA. *Defined daily doses for animals (DDDvet) and defined course doses for animals (DCDvet)*. London, UK. (2016). Available at: http://www.ema.europa.eu/docs/en_GB/document_library/Other/2016/04/WC500205410.pdf.

[29] WHO. Guidelines for ATC classification and DDD assignment. Geneva: WHO (2022).

[30] EMA. *European surveillance of veterinary antimicrobial consumption (ESVAC)*. Data Collection Protocol, (2021).

[31] Firth CL Käsbohrer A Schleicher C Fuchs K Egger-Danner C Mayerhofer M. Antimicrobial consumption on Austrian dairy farms: an observational study of udder disease treatments based on veterinary medication records. PeerJ. (2017) 5:e4072. doi: 10.7717/peerj.4072 29158993 PMC5694652

[32] EMA. *Revised ESVAC reflection paper on collecting data on consumption of antimicrobial agents per animal species, on technical units of measurement and indicators for reporting consumption of antimicrobial agents in animals*, pp. 1–29. (2013). Available at: https://www.ema.europa.eu/en/documents/scientific-guideline/revised-european-surveillance-veterinary-antimicrobial-consumption-esvac-reflection-paper-collecting_en.pdf.

[33] Firth C Käsbohrer A Egger-Danner C Fuchs K Pinior B Roch F-F. Comparison of defined course doses (DCDvet) for blanket and selective antimicrobial dry cow therapy on conventional and organic farms. Animals. (2019) 9:707. doi: 10.3390/ani9100707 31547125 PMC6826441

[34] EURL-AR. *LABORATORY PROTOCOL quantification of ESBL/AmpC-producing Escherichia coli in caecal content and fresh meat samples*. EURL-AR, (2019).

[35] European Commission. *Commission implementing decision (EU) 2020/1729 of 17 November 2020 on the monitoring and reporting of antimicrobial resistance in zoonotic and commensal bacteria and repealing implementing decision 2013/652/EU*. European Commission, pp. 8–21. (2020).

[36] EUCAST. *The European committee on antimicrobial susceptibility testing. Breakpoint tables for interpretation of MICs and zone diameters. Version 11*. (2021). Available at: http://www.eucast.org.

[37] EFSA/ECDC. The European Union summary report on antimicrobial resistance in zoonotic and indicator bacteria from humans, animals and food in 2017/2018. EFSA J. (2020) 18:e06007. doi: 10.2903/j.efsa.2020.6007, PMID: 32874244 PMC7448042

[38] EMA. *Categorisation of antibiotics in the European Union*. European Medical Agency, pp. 1–73. (2019).

[39] Firth CL Käsbohrer A Pless P Koeberl-Jelovcan S Obritzhauser W. Analysis of antimicrobial use and the presence of antimicrobial-resistant Bacteria on Austrian dairy farms–a pilot study. Antibiotics. (2022) 11:124. doi: 10.3390/antibiotics11020124 35203728 PMC8868072

[40] Pol M Ruegg PL. Treatment practices and quantification of antimicrobial drug usage in conventional and organic dairy farms in Wisconsin. J Dairy Sci. (2007) 90:249–61. doi: 10.3168/jds.S0022-0302(07)72626-7, PMID: 17183093

[41] Stevens M Piepers S Supré K Dewulf J De VS. Quantification of antimicrobial consumption in adult cattle on dairy herds in Flanders, Belgium, and associations with udder health, milk quality, and production performance. J Dairy Sci. (2016) 99:2118–30. doi: 10.3168/jds.2015-10199 26778315

[42] Kuipers A Koops WJ Wemmenhove H. Antibiotic use in dairy herds in the Netherlands from 2005 to 2012. J Dairy Sci. (2016) 99:1632–48. doi: 10.3168/jds.2014-8428, PMID: 26709178

[43] More SJ Clegg TA Mccoy F. The use of national-level data to describe trends in intramammary antimicrobial usage on Irish dairy farms from 2003 to 2015. J Dairy Sci. (2017) 100:6400–13. doi: 10.3168/jds.2016-12068, PMID: 28624279

[44] Zuliani A Lora I Brščić M Rossi A Piasentier E Gottardo F. Do dairy farming systems differ in antimicrobial use? Animals. (2020) 10:1–10. doi: 10.3390/ani10010047 PMC7023443 31881675

[45] Rinderzucht Austria. *Jahresbericht 2021. 2022, Jahresbericht 2020*, pp. 299–385. (2021).

[46] Statistik Austria. *Biologische Landwirtschaft*. (2021). Available at: https://www.statistik.at/statistiken/land-und-forstwirtschaft/land-und-forstwirtschaftliche-produktionsmethoden/biologische-landwirtschaft.

[47] Postma M Sjölund M Collineau L Lösken S Stärk KDC Dewulf J. Assigning defined daily doses animal: a European multi-country experience for antimicrobial products authorized for usage in pigs*. J Antimicrob Chemother. (2015) 70:294–302. doi: 10.1093/jac/dku347, PMID: 25223972

[48] Kyselková M Jirout J Vrchotová N Schmitt H Elhottová D. Spread of tetracycline resistance genes at a conventional dairy farm. Front Microbiol. (2015) 6:6. doi: 10.3389/fmicb.2015.00536 26074912 PMC4448040

[49] Di Cesare A Eckert EM Teruggi A Fontaneto D Bertoni R Callieri C. Constitutive presence of antibiotic resistance genes within the bacterial community of a large subalpine lake. Mol Ecol. (2015) 24:3888–900. doi: 10.1111/mec.13293, PMID: 26118321

[50] Salerno B Cornaggia M Sabatino R Di Cesare A Furlan M Barco L. Calves as Main reservoir of antibiotic resistance genes in dairy farms. Front Public Health. (2022) 10:918658. doi: 10.3389/fpubh.2022.918658 35795698 PMC9251204

[51] Feng X Littier HM Knowlton KF Garner E Pruden A. The impacts of feeding milk with antibiotics on the fecal microbiome and antibiotic resistance genes in dairy calves. Can J Anim Sci. (2020) 100:69–76. doi: 10.1139/cjas-2018-0202

[52] Schmid A Hörmansdorfer S Messelhäusser U Käsbohrer A Sauter-Louis C Mansfeld R. Prevalence of extended-spectrum β-lactamase-producing *Escherichia coli* on Bavarian dairy and beef cattle farms. Appl Environ Microbiol. (2013) 79:3027–32. doi: 10.1128/AEM.00204-13, PMID: 23455336 PMC3623142

[53] Weber LP Dreyer S Heppelmann M Schaufler K Homeier-Bachmann T Bachmann L. Prevalence and risk factors for ESBL/AmpC-*E. coli* in pre-weaned dairy calves on dairy farms in Germany. Microorganisms. (2021) 9:1–12. doi: 10.3390/microorganisms9102135 PMC8539614 34683456

[54] Massé J Lardé H Fairbrother JM Roy J Francoz D Dufour S. Prevalence of antimicrobial resistance and characteristics of *Escherichia coli* isolates from fecal and manure pit samples on dairy farms in the province of Québec. Front Vet Sci. (2021) 8:654125. doi: 10.3389/fvets.2021.654125, PMID: 34095273 PMC8175654

[55] Heuvelink AE Gonggrijp MA Buter RGJ Ter Bogt-Kappert CC van Schaik G Velthuis AGJ. Prevalence of extended-spectrum and AmpC β-lactamase-producing *Escherichia coli* in Dutch dairy herds. Vet Microbiol. (2019) 232:58–64. doi: 10.1016/j.vetmic.2019.04.005, PMID: 31030845

[56] Ohnishi M Okatani AT Esaki H Harada K Sawada T Murakami M. Herd prevalence of Enterobacteriaceae producing CTX-M-type and CMY-2 β-lactamases among Japanese dairy farms. J Appl Microbiol. (2013) 115:282–9. doi: 10.1111/jam.12211 23551813

[57] EFSA/ECDC. The European Union summary report on antimicrobial resistance in zoonotic and indicator bacteria from humans, animals and food in 2018/2019. EFSA J. (2021) 19. Available from::e06490. doi: 10.2903/j.efsa.2021.6490, PMID: 33868492 PMC8040295

[58] MacFadden DR McGough SF Fisman D Santillana M Brownstein JS. Antibiotic resistance increases with local temperature. Nat Clim Chang. (2018) 8:510–4. doi: 10.1038/s41558-018-0161-6, PMID: 30369964 PMC6201249

[59] ECDC/EFSA/EMA. Third joint inter-agency report on integrated analysis of consumption of antimicrobial agents and occurrence of antimicrobial resistance in bacteria from humans and food-producing animals in the EU/EEA. EFSA J. (2021) 19:e06712. doi: 10.2903/j.efsa.2021.6712, PMID: 34221148 PMC8243991

[60] Springer HR Denagamage TN Fenton GD Haley BJ Van Kessel JAS Hovingh EP. Antimicrobial resistance in fecal *Escherichia coli* and *Salmonella enterica* from dairy calves: a systematic review. Foodborne Pathog Dis. (2019) 16:23–34. doi: 10.1089/fpd.2018.2529 30481058

[61] Cao H. Antimicrobial resistance of Salmonella and *E. coli* from Pennsylvania dairy herds. College Park: University of Maryland (2015).

[62] Dolejská M Šenk D Čížek A Rybaříková J Sychra O Literák I. Antimicrobial resistant *Escherichia coli* isolates in cattle and house sparrows on two Czech dairy farms. Res Vet Sci. (2008) 85:491–4. doi: 10.1016/j.rvsc.2008.03.007, PMID: 18471838

[63] Jarrige N Cazeau G Bosquet G Bastien J Benoit F Gay E. Effects of antimicrobial exposure on the antimicrobial resistance of *Escherichia coli* in the digestive flora of dairy calves. Prev Vet Med. (2020) 185:105177. doi: 10.1016/j.prevetmed.2020.105177, PMID: 33181469

[64] Waade J Seibt U Honscha W Rachidi F Starke A Speck S. Multidrug-resistant enterobacteria in newborn dairy calves in Germany. PLoS One. (2021) 16:e0248291. doi: 10.1371/journal.pone.0248291, PMID: 33711073 PMC7954297

[65] Heinemann C Leubner CD Hayer JJ Steinhoff-Wagner J. Hygiene management in newborn individually housed dairy calves focusing on housing and feeding practices. J Anim Sci. (2021) 99. doi: 10.1093/jas/skaa391, PMID: 33279999 PMC7799592

[66] Pereira RVV Carroll LM Lima S Foditsch C Siler JD Bicalho RC. Impacts of feeding preweaned calves milk containing drug residues on the functional profile of the fecal microbiota. Sci Rep. (2018) 8:554. doi: 10.1038/s41598-017-19021-2 29323259 PMC5764986

[67] Tempini PN Aly SS Karle BM Pereira RV. Multidrug residues and antimicrobial resistance patterns in waste milk from dairy farms in Central California. J Dairy Sci. (2018) 2018:179–13. doi: 10.21423/aabppro20173319 30126599

[68] Brunton LA Reeves HE Snow LC Jones JR. A longitudinal field trial assesing the impact of feeding waste milk containing antibiotic residues on the prevalence of ESBL-producing *Escherichia coli* in calves. Prev Vet Med. (2014) 117:403–12. doi: 10.1016/j.prevetmed.2014.08.005, PMID: 25172121

[69] Randall L Heinrich K Horton R Brunton L Sharman M Bailey-Horne V. Detection of antibiotic residues and association of cefquinome residues with the occurrence of extended-Spectrum β-lactamase (ESBL)-producing bacteria in waste milk samples from dairy farms in England and Wales in 2011. Res Vet Sci. (2014) 96:15–24. doi: 10.1016/j.rvsc.2013.10.009, PMID: 24314891

[70] Gonggrijp MA Velthuis AGJ Heuvelink AE van den Heuvel KWH ter Bogt-Kappert CC Buter GJ. Prevalence of extended-spectrum and AmpC β-lactamase-producing *Escherichia coli* in young calves on Dutch dairy farms. J Dairy Sci. (2023) 106:4257–65. doi: 10.3168/jds.2022-22362, PMID: 37028968

[71] MARAN. *Monitoring of antimicrobial resistance and antibiotic usage in animals in the Netherlands in 2021*. MARAN, pp. 1–286. (2022).

[72] Rell J Wunsch N Home R Kaske M Walkenhorst M Vaarst M. Stakeholders’ perceptions of the challenges to improving calf health and reducing antimicrobial use in Swiss veal production. Prev Vet Med. (2020) 179:104970. doi: 10.1016/j.prevetmed.2020.104970, PMID: 32407997

[73] Pereira RV Siler JD Ng JC Davis MA Grohn YT Warnick LD. Effect of on-farm use of antimicrobial drugs on resistance in fecal *Escherichia coli* of preweaned dairy calves. J Dairy Sci. (2014) 97:7644–54. doi: 10.3168/jds.2014-8521, PMID: 25306279 PMC4351791

[74] Maynou G Chester-Jones H Bach A Terré M. Feeding pasteurized waste milk to preweaned dairy calves changes fecal and upper respiratory tract microbiota. Front Vet Sci. (2019) 6:159. doi: 10.3389/fvets.2019.00159 31245388 PMC6562338

[75] Dong L Meng L Liu H Wu H Hu H Zheng N. Effect of therapeutic administration of β-lactam antibiotics on the bacterial community and antibiotic resistance patterns in milk. J Dairy Sci. (2021) 104:7018–25. doi: 10.3168/jds.2020-20025, PMID: 33741154

[76] Gonggrijp MA Hage JJ Heuvelink AE Velthuis A. Prevalence and risk factors for extended-spectrum β -lactamase or AmpC-producing *Escherichia coli* in organic dairy herds in the Netherlands. J Dairy Sci. (2017) 100:562–71. doi: 10.3168/jds.2016-11839 27865491

[77] Shrestha RD Agunos A Gow SP Deckert AE Varga C. Associations between antimicrobial resistance in fecal *Escherichia coli* isolates and antimicrobial use in Canadian Turkey flocks. Front Microbiol. (2022) 13:1–16. doi: 10.3389/fmicb.2022.954123 PMC9372513 35966666

[78] Munita JM Arias CA. Mechanisms of antibiotic resistance. Compend Contin Educ Pract Vet. (2016) 23:464–72. doi: 10.1128/microbiolspec.VMBF-0016-2015

[79] Turner A Schubert H Puddy EF Sealey JE Gould VC Cogan TA. Factors influencing the detection of antibacterial-resistant *Escherichia coli* in faecal samples from individual cattle. J Appl Microbiol. (132) 132:2633–41. doi: 10.1111/jam.15419 34923720

