# Supplementary material for: Antimicrobial resistance and its relationship with antimicrobial use on Austrian dairy farms
Source: Front Vet Sci. 2023 Jul 21;10:1225826. doi: 10.3389/fvets.2023.1225826 (PMC10403287; doi:10.3389/fvets.2023.1225826)
Supplement: Supplementary file 1 [file Table_1.DOCX]

# Supplementary Material

**Table S1.** Antibiotic classes, antibiotics tested and epidemiological cut-off values for panel EUVSEC3

| **Antibiotic class** | **Antibiotic** | **Cut-off value (mg/L)** |
| --- | --- | --- |
| Aminoglycoside | Amikacin | > 8 |
| Penicillin | Ampicillin | > 8 |
| Macrolide | Azithromycin | > 16 |
| Cephalosporin | Cefotaxime | > 0.25 |
| Cephalosporin | Ceftazidime | > 0.5 |
| Phenicol | Chloramphenicol | > 16 |
| Fluoroquinolone | Ciprofloxacin | > 0.064 |
| Polymyxin | Colistin | > 2 |
| Aminoglycoside | Gentamicin | > 2 |
| Carbapenem | Meropenem | > 0.125 |
| Chinolone | Nalidixic acid | > 8 |
| Folate pathway antagonist (Sulfonamide) | Sulfamethoxazole | > 64 |
| Tetracycline | Tetracycline | > 8 |
| Glycylcycline | Tigecycline | > 0.5 |
| Folate pathway antagonist (Dihydrofolate reductase inhibitor) | Trimethoprim | > 2 |

**Table S2.** Antibiotic classes, antibiotics tested and epidemiological cut-off values for panel EUVSEC2.

| **Antibiotic class** | **Antibiotic** | **Cut-off value (mg/L)** |
| --- | --- | --- |
| Cephalosporin | Cefepime | > 0.125 |
| Cephalosporin | Cefotaxime | > 0.25 |
| ß-lactam combination agent | Cefotaxime-clavulanic acid | > 0.25 |
| Cephamycin | Cefoxitin | > 8 |
| Cephalosporin | Ceftazidime | > 0.5 |
| ß-lactam combination agent | Ceftazidim-clavulanic acid | > 0.5 |
| Carbapenem | Ertapenem | 0.03 |
| Carbapenem | Imipenem | > 0.5 |
| Carbapenem | Meropenem | > 0.125 |
| Penicillin | Temocillin | > 16 |
